# A Cross-sectional Conceptual Replication and Longitudinal Evaluation of the PANSS-Autism-Severity-Score Measure Suggests it Does Not Capture Autistic Traits in Individuals With Psychosis

**DOI:** 10.1093/schbul/sbad161

**Published:** 2023-11-22

**Authors:** Katharine Chisholm, Frederike Schirmbeck, Amy E Pinkham, Noah J Sasson, Claudia J P Simons, Lieuwe de Haan, Philip D Harvey, David L Penn, Tim Ziermans

**Affiliations:** School of Psychology, Institute of Health and Neurodevelopment, Aston University, Aston St, Birmingham, B4 7ET, UK; Department of Psychiatry, Amsterdam University Medical Center, Meibergdreef, University of Amsterdam, Amsterdam, The Netherlands; Arkin Institute for Mental Health, Amsterdam, The Netherlands; Department of Psychology, School of Behavioral and Brain Sciences, The University of Texas at Dallas, Richardson, TX, USA; Department of Psychology, School of Behavioral and Brain Sciences, The University of Texas at Dallas, Richardson, TX, USA; Department of Psychiatry and Neuropsychology, Maastricht University Medical Center, School for Mental Health and Neuroscience, Maastricht, The Netherlands; GGzE Institute for Mental Health Care, Eindhoven, The Netherlands; Department of Psychiatry, Amsterdam University Medical Center, Meibergdreef, University of Amsterdam, Amsterdam, The Netherlands; Arkin Institute for Mental Health, Amsterdam, The Netherlands; Department of Psychiatry and Behavioral Sciences, University of Miami, Miller School of Medicine, Miami, FL, USA; Research Service, Miami VA Healthcare System, Miami, USA; Department of Psychology and Neuroscience, University of North Carolina, Chapel Hill, USA; School of Behavioural and Health Sciences, Australian Catholic University, Melbourne, Victoria, Australia; Department of Psychology, Dutch Autism & ADHD Research Center, University of Amsterdam, Amsterdam, The Netherlands

**Keywords:** “Schizophrenia spectrum disorders”, “Autistic traits”, “Measure validation”, Psychosis, PANSS

## Abstract

**Background:**

Autism and psychosis co-occur at elevated rates, with implications for clinical outcomes, functioning, and suicidality. The PANSS-Autism-Severity-Score (PAUSS) is a measure of autism trait severity which has not yet been validated externally or longitudinally.

**Study Design:**

Participants were derived from the GROUP and SCOPE datasets. Participants included 1448 adults with schizophrenia spectrum disorder (SSD), 800 SSD-siblings, 103 adults diagnosed with an autistic spectrum condition (ASC), and 409 typically-developing controls (TC). Analyses from the original validation study were conducted with SSD participants, and extended into ASC, SSD-sibling, and TC participants. Test–retest reliability of the PAUSS at 2-weeks and long-term stability 3 and 6-years was also examined.

**Study Results:**

Results differed in important ways from the original validation. SSD participants reported higher PAUSS scores than other groups, with only a fraction of ASC participants scoring as “PAUSS-Autistic.” Cronbach’s alpha was acceptable for the SSD cohort only. Two-week stability of the PAUSS was fair to good for all PAUSS scores. Long-term stability was poor for most PAUSS items but fair for total PAUSS score.

**Conclusions:**

Results suggest that the PAUSS does not appear appropriate for assessing autism, with the low rate of PAUSS-Autistic in the ASC population suggesting the PAUSS may not accurately reflect characteristics of autism. The relative lack of long-term stability is cause for concern and suggestive that the PAUSS is capturing features of psychosis rather than autism traits.

## Introduction

Autism and psychosis co-occur at elevated rates,^1–6^ with implications for clinical outcomes, functioning, and suicidality.^7–11^ Traditionally, research has concentrated on *diagnostic* co-occurrence,^5,6^ however, more recent work has examined the overlap continuously, revealing a high prevalence of subclinical autistic traits within psychosis.^7,8^ Importantly, autistic traits, even in the absence of an autism diagnosis, have been linked to poorer clinical outcomes, including increased suicidality.^8,9^ Better understanding the potential role of autistic traits within psychosis may therefore help to improve treatment, develop interventions, and prevent negative outcomes like suicide.

The measurement of autistic traits in psychosis populations is complex and validated instruments do not exist. Assessments of autism such as the Autism Diagnostic Observation Schedule (ADOS)^12^ focus on diagnostic classification rather than autistic trait measurement, require extensive training, and are time consuming to administer, which affects healthcare access and costs. As an easily implemented alternative, many researchers have begun using the PANSS-Autism-Severity-Score (PAUSS),^13^ a measure of autism trait severity derived from the Positive and Negative Syndrome Scale (PANSS).^14^ The PANSS is routinely used to assess symptom severity in schizophrenia spectrum disorders (SSD). To create a PAUSS score, items from the PANSS which are considered indicative of autistic-like behavior (covering all autistic domains within the DSM-IV-TR) are extracted and summed together (see Box 1). The widespread use of the PANSS has made PAUSS scores straightforward to calculate in both archival and current datasets.

Kästner et al,^13^ developed and assessed the PAUSS by examining its validity within a large Autism Spectrum Condition (ASC; *n* = 265) and SSD sample (*n* = 1156). Within ASC, the PAUSS demonstrated good convergent validity with the ADOS^12^ and high criterion-related validity differentiating autistic participants and a “disease-control” sample. The PAUSS demonstrated high internal reliability within SSD and significant differences in education, treatment, pre-morbid intelligence, functioning, psychotic symptoms, and age between “PAUSS-Autistic Schizophrenia” (PAUSS total ≥30) and “PAUSS-Non-Autistic Schizophrenia” (≤10).^13^ Subsequent validation has reported high internal reliability in both ASC (*n* = 33) and SSD (*n* = 26) samples, medium to high convergent validity in SSD, inconsistent convergent validity in ASC, and found that PAUSS total score is significantly associated with global functioning in SSD.^15^ A lower cut-off of >17 for PAUSS-Autistic was suggested, with PAUSS-Autistic participants (*n* = 13) scoring higher on the ADOS than PAUSS-Non-Autistic participants (*n* = 13), but lower than the ASC group. In a separate study^16^ of 75 SSD participants, those identified as autistic on the ADOS (*n* = 14) and ADI-R (*n* = 9) had higher PAUSS scores and functional deficits.

Although the PAUSS is gaining traction with researchers,^17^ it has not yet been fully validated externally. Other than the development paper, validation samples have been small, and cut-offs remain undetermined. Similarly, in ASC populations, ADOS and related measures have had mixed convergent validity.

The PAUSS has also not been investigated in high-risk groups such as siblings of those with psychosis. Siblings may share SSD phenotypes^18–20^ and have up to a 10-fold increased risk of developing SSD.^21^ Research into siblings therefore presents an invaluable resource for investigating state versus trait SSD markers, and identifying potential endophenotypes^22^ within a cohort uncomplicated by anti-psychotic medication or severity of illness.

Most importantly, the PAUSS has not been validated longitudinally. Autism can be considered a stable and trait-like diagnosis and identity,^23,24^ whereas SSD symptomatology is less stable.^25,26^ Negative psychotic symptoms show less variation than positive symptoms, however both change over time.^26^ The PANSS, which the PAUSS is derived from, was designed to quantify state psychosis during the past week,^14^ rather than capturing trait-like characteristics. Longitudinal validation of the PAUSS is therefore vital.

The present study has two aims. First, we sought to conceptually replicate the analyses from the original PAUSS validation paper. Specifically, we examined internal consistency, convergent validity, and the utility of PAUSS cut-offs across (1) a large cohort of individuals with SSD with a broader age range than the original study, (2) autistic adults (ASC), (3) typically-developing controls (TC), and (4) SSD-siblings. We also investigated the utility of the PAUSS in predicting ASC vs TC group membership. Second, we examined the short and long-term PAUSS stability over 2-week, 3- and 6-year intervals. We hypothesized that SSD-siblings would show higher PAUSS scores than TC, and lower PAUSS scores than SSD and ASC cohorts. Since the PANSS is a state measure, we projected high short-term but only moderate long-term PAUSS stability.

## Method

### Sample Characteristics

Participants were derived from the Genetic Risk and Outcome of Psychosis (GROUP; 1022 SSD, 700 SSD-sibling, and 409 TC participants),^27^ the Social Cognition Psychometric Evaluation (SCOPE; 426 SSD participants),^28,29^ and SCOPE in Autism (SCOPE-A; 103 ASC participants)^30,31^ study datasets. GROUP and SCOPE are both multisite longitudinal observational studies.

GROUP has three data collection waves: baseline, 3- and 6-years. This study utilized SSD data from all three waves. Sibling and TC data were taken from the 3-year wave, which had the most complete data for SSD-siblings and controls. SSD-siblings and TCs who transitioned to psychosis were excluded.

SCOPE data which was utilized by this study consisted of SSD data from baseline and 2-week follow-up. SCOPE-A was a companion project to SCOPE; ASC participants completed measures once.

**Fig. 1.**
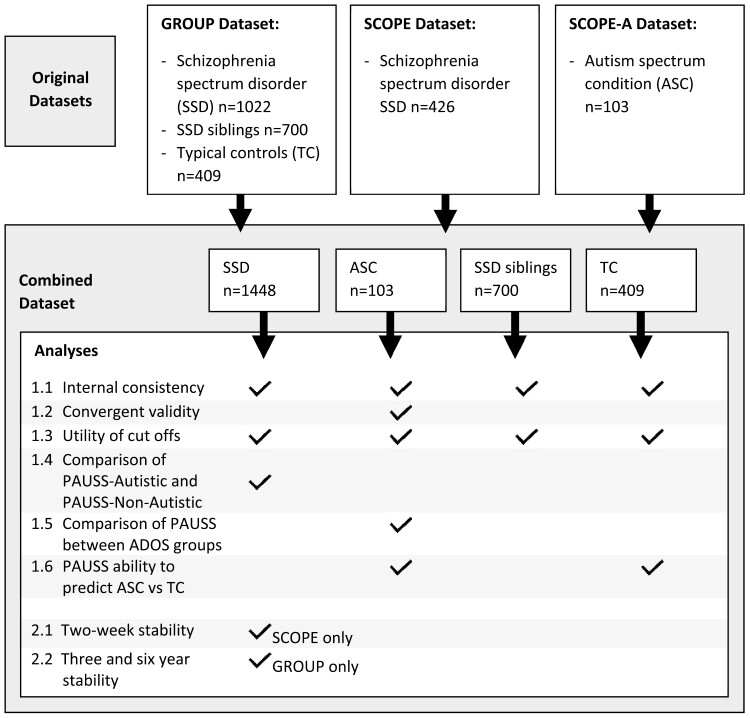
Original datasets, Combined Dataset, and involvement with analyses.

GROUP and SCOPE data were entered into a Combined Dataset (figure 1), which consisted of 1448 SSD, 103 ASC, 700 SSD-siblings, and 409 TC with complete PAUSS data. For additional information about the GROUP, SCOPE, and SCOPE-A datasets see Supplementary material S1.

### Materials

#### Psychotic Symptoms

The PANSS^14^ was used to measure psychotic symptoms across all participants. The PANSS contains 30 interviewer-rated items, is well-validated, and assesses the severity of positive, negative, and general psychotic symptoms occurring over the past week. Higher scores on the PANSS indicate greater psychotic symptom severity.

#### Autistic Behaviors

The PAUSS^13^ was used to measure autistic traits across all participants. The PAUSS consists of 8 items from the PANSS (Box 1). Higher scores on the PAUSS indicate a higher number of autistic-like behaviors.

Box 1. **PAUSS Items Taken Verbatim from the PANSS**

**Table:** 

Autism behavioral clusters within the DSM-IV-TR	PAUSS item (original PANSS number)	Original description from the PANSS General Rating Instructions
Differences in social interaction	Blunted Affect (N1)	“Diminished emotional responsiveness as characterised by a reduction in facial expression, modulation of feelings and communicative gestures”
	Poor Rapport (N3)	“Lack of interpersonal empathy, openness in conversation and sense of closeness, interest or involvement with the interviewer”
	Passive/Apathetic Social Withdrawal (N4)	“Diminished interest and initiative in social interactions due to passivity, apathy, anergy or avolition”
Differences in communication	Difficulty in Abstract Thinking (N5)	“Impairment in the use of the abstract-symbolic mode of thinking”
	Lack of Spontaneity and Flow of Conversation (N6)	“Reduction in the normal flow of communication associated with apathy, avolition, defensiveness or cognitive deficit”
Restricted and repetitive patterns of behaviour	Stereotyped thinking (N7)	“Decreased fluidity, spontaneity and flexibility of thinking, as evidenced in rigid, repetitious or barren thought content”
	Mannerisms and Posturing (G5)	“Unnatural movements or posture as characterised be an awkward, stilted, disorganized, or bizarre appearance”
	Preoccupation (G15)	“Absorption with internally generated thoughts and feelings and with autistic experiences to the detriment of reality orientation and adaptive behaviour”

*Note*: PANSS, Positive and Negative Syndrome Scale; PAUSS, PANSS-Autism-Severity-Score.

#### Autism Diagnosis

The ADOS-2^32^ was used to establish autism diagnosis within the Autistic cohort. The ADOS-2 is a well-validated, gold standard tool for confirming autism diagnosis. Scores of 7–11 are classified as autism spectrum and scores of >11 as autism. All included autistic participants in the current study scored 7 or higher.

#### Intelligence Quotient (IQ)

The Wechsler Abbreviated Scale of Intelligence (WASI^33^) was used to estimate intelligence quotient (IQ) within the ASC sample. The WASI is a well-validated measure of IQ, with higher scores indicating higher estimated IQ. Participants completed verbal and matrix reasoning sections. Raw verbal and non-verbal scores were converted to t-scores, which were used to calculate standardized full-scale IQ.

### Analysis

All analyses were conducted with SPSS version 26. Initial analyses included a series of Kruskal–Wallis and chi-square tests to investigate differences in demographics and complete PANSS scores between the four participant groups. Difference in PANSS severity between our SSD sample and the sample reported in Kästner et al, was also examined via a series of one-sample *t*-tests.

Main analyses (figure 1) first sought to conceptually replicate, where possible for the current dataset, Kästner et al’s^13^ original PAUSS paper. This included examining:

1.1 The internal consistency, via Cronbach’s α and inter-item correlations, of the PAUSS items in all participant groups.
1.2 The convergent validity, via Spearmans rho intercorrelations, of the PAUSS total with ADOS total, age, and WASI IQ, as well as intercorrelations of individual PAUSS items with the ADOS total, in the ASC sample.
1.3 The utility of the PAUSS cut-offs; number of participants from each group (SSD, ASC, SSD-Siblings, TC) falling into Kästner et al’s defined cut-offs (based on 1st and last PAUSS total percentile from their SSD cohort) for PAUSS-Autistic and PAUSS-Non-Autistic was calculated. Additionally, following the same method as Kästner et al (1st and last PAUSS total percentile from *our* SSD cohort) cut-offs were computed, and number of participants from each group within these new cut-offs were examined.
1.4 PAUSS-Autistic and PAUSS-Non-Autistic SSD participants were then compared on participant characteristics and PANSS scores, via a series of Mann–Whitney *U* and Chi-Square tests. For analyses relating to aim 1.4 only, PAUSS items were excluded when calculating all PANSS subscales. For all other analyses, the PANSS was scored as per scoring instructions from the original PANSS development paper.^14^
1.5 Comparison of PAUSS score between the ADOS groups (autism > 11/autism spectrum 7–11) within the ASC sample was examined via a Mann–Whitney *U* test.
1.6 The ability of the PAUSS to predict ASC group membership compared to TC group membership was investigated via receiver operator curve (ROC) analysis.

To address the second aim of the paper investigating the short- and long-term stability of the PAUSS, short (2-week) and long-term (3- and 6-year) test–retest reliability of the PAUSS in SSD participants was examined via Pearson’s *r* correlations.

Due to the high number of analyses conducted, the critical *P*-value value was set at .005.^34^ SSD participants from GROUP and SCOPE were found to differ significantly in terms of their age, gender, PANSS total, PANSS positive, PANSS negative, and PANSS general (see Supplementary material S2). Combining two diverse cohorts was seen as a strength of the analysis in aiding generalizability. For completeness, however, additional analyses including dataset as a covariate to analyses where possible, or analysing GROUP and SCOPE SSD participants separately to each other where inclusion of covariates was not possible, can be viewed in Supplementary material S3. In line with Kästner et al, age and sex were not included as covariates in the main analysis. Analyses including age and sex as covariates can be viewed in Supplementary material S4.

## Results

Within the Combined Dataset, the participant groups (SSD, ASC, SSD-siblings, TC) were found to differ significantly from each other after Bonferroni correction in terms of age, gender, PANSS total, PANSS positive, PANSS negative, PANSS general, and PAUSS total (table 1). For PANSS negative all participant groups except TC—SSD-siblings and SSD-ASC were significantly different. For age, all participant groups except SSD—SSD-siblings were significantly different. For PANSS total, general, and positive all participant groups except TC—SSD-siblings were significantly different.

**Table 1.** Sample Characteristics

	GROUP	SCOPE	Combined Dataset
Age; mean (SD)			
SSD	27.36 (7.45)	39.91 (12.48)	31.05 (10.84)
ASC	—	24.28 (6.18)	24.28 (6.18)
SSD-siblings	30.85 (7.92)	—	30.85 (7.92)
TC	34.4 (10.67)	—	34.4 (10.67)
Statistical significance between participant groups in Combined Dataset			*H*(3) = 105.43, *P* < .001, η^2^ [*H*] = 0.04
Gender; *n* male (%)			
SSD	789 (77.2)	288 (67.6)	1077 (74.4)
ASC	—	92 (89.3)	92 (89.3)
SSD-siblings	310 (44.3)	—	310 (44.3)
TC	186 (45.5)	—	186 (45.5)
Statistical significance between participant groups in Combined Dataset			X2(3, *n* = 2660) = 268.70, *P* < .001, *V* = 0.30
PANSS Total; mean (SD)			
SSD	54.41 (16.80)	62.86 (14.62)	56.97 (16.62)
ASC	—	46.02 (8.06)	46.02 (8.06)
SSD-siblings	32.29 (4.23)	—	32.29 (4.23)
TC	31.57 (2.86)	—	31.57 (2.86)
Statistical significance between participant groups in Combined Dataset			*H*(3) = 1659.12, *P* < .001, η^2^ [*H*] = 0.64
PANSS Positive; mean (SD)			
SSD	12.69 (5.31)	16.37 (5.27)	13.78 (5.55)
ASC	—	9.89 (2.98)	9.89 (2.98)
SSD-siblings	7.30 (1.00)	—	7.30 (1.00)
TC	7.27 (0.83)	—	7.27 (0.83)
Statistical significance between participant groups in Combined Dataset			*H*(3) = 1389.39, *P* < .001, η^2^ [*H*] = 0.53
PANSS Negative; mean (SD)			
SSD	13.90 (5.90)	14.29 (5.31)	14.02 (5.74)
ASC	—	12.81 (4.73)	12.81 (4.73)
SSD-siblings	7.58 (1.65)	—	7.58 (1.65)
TC	7.25 (0.68)	—	7.25 (0.68)
Statistical significance between participant groups in Combined Dataset			*H*(3) = 1346.71, *P* < .001, η^2^ [*H*] = 0.51
PANSS General; mean (SD)			
SSD	27.89 (8.40)	32.20 (7.98)	29.19 (8.51)
ASC	—	23.32 (4.81)	23.32 (4.81)
SSD-siblings	17.43 (2.63)	—	17.43 (2.63)
TC	17.05 (2.11)	—	17.05 (2.11)
Statistical significance between participant groups in Combined Dataset			*H*(3) = 1522.39, *P* < .001, η^2^ [*H*] = 0.58
PAUSS total; mean (SD)			
SSD	14.56 (5.70)	15.52 (5.21)	14.84 (5.57)
ASC	—	13.85 (4.23)	13.85 (4.23)
SSD-siblings	8.54 (1.53)	—	8.54 (1.53)
TC	8.23 (0.69)	—	8.23 (0.69)
Statistical significance between participant groups in Combined Dataset			*H*(3) = 1369.79, *P* < .001, η^2^ [*H*] = 0.51

*Note*: ASC, autism spectrum condition; PANSS, Positive and Negative Syndrome Scale; PAUSS, PANSS-Autism-Severity-Score; SSD, schizophrenia spectrum disorder; TC, typical control.

**Fig. 2.**
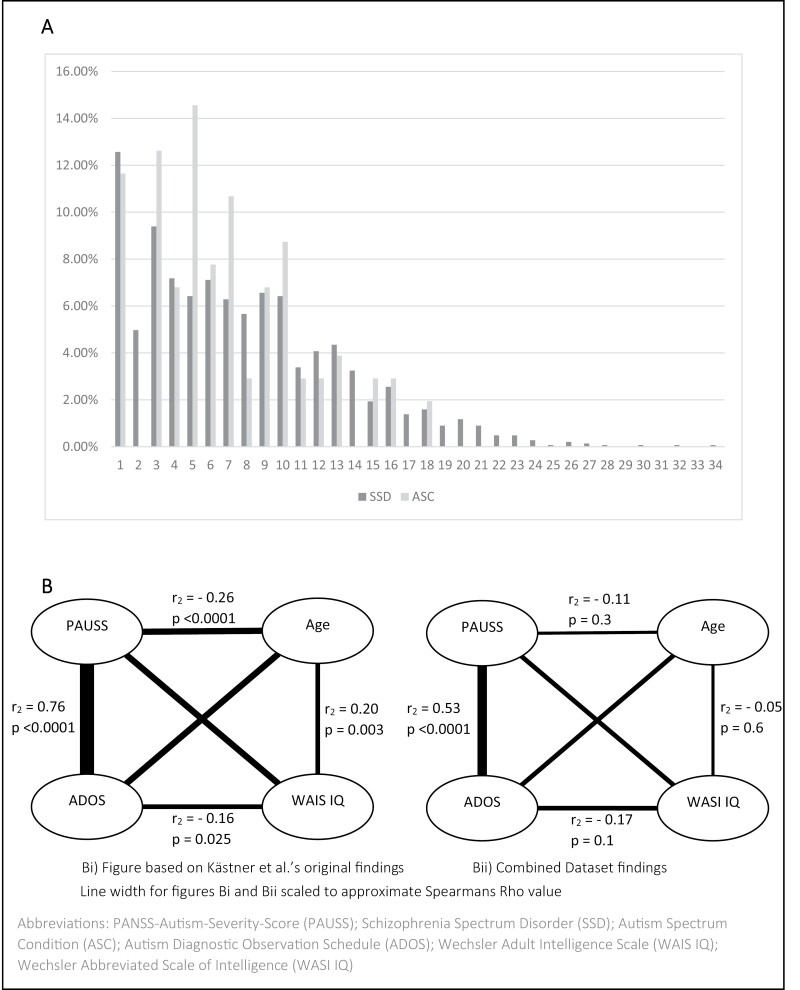
SSD and ASC percentage distribution of PAUSS total score (A) and intercorrelations of the PAUSS total and ADOS total with age and WAIS IQ (Kästner et al)/WASI IQ (SCOPE dataset) in ASC (B).

SSD and ASC participant groups both scored significantly higher on the PAUSS compared to TC and SSD-sibling groups. 86.3% of TC and 79.6% of SSD-siblings scored the lowest possible PAUSS score of 8, compared to just 12.6% of the SSD and 11.7% of the ASC cohorts. Distribution of PAUSS scores within the SSD and ASC cohorts can be seen in figure 2A.

Compared to SSD participants within our Combined Dataset, the SSD sample reported in Kästner et al, experienced comparable positive psychotic symptoms (*t*(1432) = 0.97, *P* = .3, *d* = 0.03), but significantly more severe negative (*t*(1447) = 27.02, *P* < .001, *d* = 0.71), general (*t*(1416) = 25.91, *P* < .001, *d* = 0.69), and total (*t*(1403) = 22.13, *P* < .001, *d* = 0.59) psychotic symptoms.

### Aim One: To Conceptually Replicate the Analyses from the Original PAUSS Validation Paper (Kästner et al)

#### Internal Consistency of PAUSS Items Across Cohorts

Kästner et al, reported a good Cronbach’s α of .86 within their SSD cohort. Internal consistency was found to be acceptable within our SSD cohort only (α = .76; driven by a good α within the GROUP cohort and a questionable α within the SCOPE cohort, see Supplementary material S3), with questionable, poor, and unacceptable consistency found, respectively, within the SSD-sibling (α = .68; driven by an acceptable α within male participants and an unacceptable α within female participants, see Supplementary material S4), ASC (α = .58 driven by a questionable α within female participants and a poor α within male participants, see Supplementary material S4), and TC (α = .30 driven by an acceptable α within female participants and an unacceptable α within male participants, see Supplementary material S4). Inter-item correlations can be seen in table 2. Small, non-significant, or negative inter-item correlations were found between several PAUSS items within all cohorts except for SSD.

**Table 2.** Item to Item Spearman’s Rho Intercorrelation Matrix for Individual PAUSS Items and ADOS

	Blunted Affect (N1)	Poor Rapport (N3)	Social Withdrawal (N4)	Abstract Thinking (N5)	Lack of Spontaneity (N6)	Stereotyped Thinking (N7)	Mannerism (G5)	Preoccupation (G15)
Poor rapport (PANSS N3)								
Kästner et al, SSD	**0.67**	—						
SSD	**0.56**	—						
ASC	**0.52**	—						
SSD-siblings	**0.50**	—						
TC	−0.02	—						
Social withdrawal (PANSS N4)								
Kästner et al, SSD	**0.57**	**0.58**	—					
SSD	**0.44**	**0.42**	—					
ASC	**0.45**	0.28	—					
SSD-siblings	**0.48**	0.35	—					
TC	−0.02	0.15	—					
Abstract thinking (PANSS N5)								
Kästner et al, SSD	**0.47**	**0.45**	0.303	—				
SSD	0.16	0.16	0.21	—				
ASC	0.21	0.17	0.15	—				
SSD-siblings	0.18	0.18	0.09	—				
TC	−0.04	0.05	−0.03	—				
Conversation (PANSS N6)								
Kästner et al, SSD	**0.58**	**0.60**	**0.57**	**0.43**	—			
SSD	**0.61**	**0.66**	**0.42**	0.23	—			
ASC	**0.50**	**0.50**	0.37	0.13	—			
SSD-siblings	**0.55**	**0.57**	**0.42**	0.18	—			
TC	0.19	0.12	0.12	0.22	—			
Stereotyped thinking (PANSS N7)								
Kästner et al, SSD	**0.41**	**0.44**	0.33	**0.40**	0.25	—		
SSD	0.24	0.30	0.28	0.26	0.25	—		
ASC	−0.02	−0.03	0.03	0.25	0.05	—		
SSD-siblings	0.24	0.27	0.33	0.19	0.26	—		
TC	−0.02	0.20	0.20	−0.03	0.15	—		
Mannerism (PANSS G5)								
Kästner et al, SSD	0.26	0.24	0.22	0.25	0.14	0.32	—	
SSD	0.30	0.25	0.18	0.16	0.26	0.30	—	
ASC	−0.03	0.14	−0.10	0.06	−0.08	−0.10	—	
SSD-siblings	0.23	0.32	0.37	0.09	0.31	0.26	—	
TC	−0.02	0.20	−0.01	−0.03	−0.02	−0.01	—	
Preoccupation (PANSS G15)								
Kästner et al, SSD	**0.49**	**0.56**	**0.50**	0.39	0.36	**0.52**	0.29	—
SSD	0.16	0.24	0.21	0.15	0.15	**0.49**	0.21	—
ASC	−0.16	−0.15	−0.11	0.12	−0.14	**0.41**	0.11	—
SSD-siblings	0.21	0.24	0.28	0.04	0.35	0.24	0.11	—
TC	0.13	0.17	0.18	−0.03	0.13	**0.67**	−0.01	—
ADOS (ASC cohort only)								
Kästner et al, ASC	**0.6–0.7**	**0.5–0.6**	**0.6–0.7**	**0.4–0.5**	**0.6–0.7**	**0.4–0.5**	**0.6–0.7**	**0.6–0.7**
ASC	0.37	0.27	**0.42**	0.30	**0.40**	0.21	−0.01	0.04

*Note*: ADOS, autism diagnostic observation schedule; ASC, autism spectrum condition; PANSS, Positive and Negative Syndrome Scale; SSD, schizophrenia spectrum disorder; TC, typical control.

—SSD (*n* = 1448): all correlations were significant at the *P* < .001 level.

—For ASC (*n* = 103): correlations >.27 were significant at the *P* < .005 level, and all correlations >.36 were significant at the *P* < .001 level

—For SSD-siblings (*n* = 700): correlations >.10 were significant at the *P* < .005 level, and all correlations >.74 were significant at the *P* < .001 level.

—For TC (*n* = 409): correlations >.14 were significant at the *P* < .005 level, and all correlations >.17 were significant at the *P* < .001 level.

#### Spearmans Rho Intercorrelations of PAUSS Total and ADOS Total Within ASC Cohort

We found correlations of a similar direction and approximately similar strength to Kästner et al’s findings (figure 2B). The correlation between the PAUSS and the ADOS was strong and positive, albeit lower than the correlation found by Kästner et al.

#### Intercorrelations of Individual PAUSS Items With the ADOS Total Within ASC Cohort

Kästner et al, report positive correlations of between .4 and .7 between all items of the PAUSS and the ADOS. We found correlations of above .4 for only two of the PAUSS items (N4; N6), with N1 additionally showing a medium positive correlation with the ADOS. N5 displayed a weak, positive correlation, with N3, N7, G15, and G5 all displaying non-significant relationships with the ADOS (table 2).

#### Comparison of PAUSS-Autistic and PAUSS-Non-*autistic Participants Across Cohorts.*

*Cut-offs derived from Kästner* et al: Within Kästner et al’s SSD dataset 14.53% were classified as PAUSS-Non-Autistic (PAUSS total ≤ 10) and 11.85% as PAUSS-Autistic (PAUSS total ≥ 30), using 1st and last PAUSS percentile derived cut-offs from their SSD participants. Within our SSD cohort 1.5% were classified as PAUSS-Autistic, and 26.9% as PAUSS-Non-Autistic. No participants within the TC and SSD-sibling cohorts were defined as PAUSS-Autistic, with 97.8% and 93.7%, classified as PAUSS-Non-Autistic, respectively. Strikingly, no ASC individuals were identified as PAUSS-Autistic and 24.3% were classified as PAUSS-Non-Autistic.

Within our SSD cohort PANSS total, positive, negative, and general differed significantly between PAUSS-Autistic and PAUSS-Non-Autistic (table 3). The significant difference between in PANSS positive symptoms was driven by female participants (see Supplementary material S4).

**Table 3.** Comparison of PAUSS-Autistic and Non-autistic Based on Kästner et al’s Original Cut-offs and Alternative Cut-offs Derived from Our SSD Cohort

	PAUSS-Autistic	PAUSS-Non-Autistic	Significance (Effect Size)
Original cut-offs			
Kästner et al, SSD *n* (%)	137 (11.85%) of 1156	168 (14.53%) of 1156	—
Combined Dataset SSD *n* (%)	21 (1.5) of 1448	390 (26.9%) of 1448	—
Age mean/median (SD)	27.24/25 (9.50)	30.1/27 (9.84)	0.1 (η^2^ = 0.006)
Gender male *n* (%)	20 (95.2)	268 (68.7)	0.007 (φ = 0.02)
PANSS Total (PAUSS items excluded) mean/median (SD)	61.26/66 (12.44)	32.66/30.50 (8.50)	<0.0001 (η^2^ = 0.11)
PANSS positive mean/median (SD)	17.19/17 (6.54)	10.92/10 (4.19)	<0.0001 (η^2^ = 0.05)
PANSS negative (PAUSS items excluded) mean/median (SD)	4.10/4 (1.45)	1.21/1 (0.52)	<0.0001 (η^2^ = 0.23)
PANSS general (PAUSS items excluded) mean/median (SD)	39.16/42 (8.19)	20.55/20 (5.29)	<0.0001 (η^2^ = 0.11)
Alternative cut-offs			
Combined Dataset SSD *n* (%)	151 (10.4%) of 1448	182 (12.6%) of 1448	—
Age mean/median (SD)	28.88/25 (10.20)	29.86/27 (9.74)	0.1 (η^2^ = 0.01)
Gender male *n* (%)	123 (81.5)	119 (65.4)	0.001 (φ = 0.03)
PANSS total (PAUSS items excluded) mean/median (SD)	55.11/54 (12.48)	30.48/28 (8.12)	<0.0001 (η^2^ = 0.61)
PANSS positive mean/median (SD)	17.05/16 (6.38)	10.09/9 (3.86)	<0.0001 (η^2^ = 0.35)
PANSS negative (PAUSS items excluded) mean/median (SD)	3.55/4 (1.15)	1.05/1 (0.26)	<0.0001 (η^2^ = 0.79)
PANSS general (PAUSS items excluded) mean/median (SD)	34.39/34 (7.42)	19.38/18 (5.14)	<0.0001 (η^2^ = 0.60)

*Note*: PANSS, Positive and Negative Syndrome Scale; PAUSS, PANSS-Autism-Severity-Score; SSD, Schizophrenia Spectrum Disorder.

#### Cut-offs *Derived* from *Our* SSD *Dataset.*

 1st and last PAUSS total percentile based on our SSD dataset were computed, suggesting alternative cut-offs of ≥24 for PAUSS-Autistic and ≤8 for PAUSS-Non-Autistic. Using these cut-offs, we found 10.4% SSD participants were classified as PAUSS-Autistic and 12.6% as PAUSS-Non-Autistic. Within the ASC cohort 4.9% were PAUSS-Autistic and 11.7% PAUSS-Non-Autistic. Within the TC and SSD-sibling cohorts no participants were classified as PAUSS-Autistic, with 86.3% and 79.6% respectively classified as PAUSS-Non-Autistic. Significant differences were found within the SSD cohort for gender, PANSS total, positive, negative, and general (table 3).

#### Comparison of PAUSS Total Between ADOS Diagnostic Groups Within ASC Cohort

Kästner et al, found the PAUSS differed significantly between all three ADOS groups (no autism, autistic spectrum, autistic) in their ASC sample. Within our ASC cohort 36 participants were classified by the ADOS as autistic (PAUSS mean = 16.67, median = 17, SD = 4.37), 65 as autistic spectrum (PAUSS mean = 12.32, median = 12, SD = 3.35), 2 had missing ADOS data, and no participants fell into the no autism category. PAUSS score of the two ADOS groups differed significantly (*P* < .0001, η^2^ = 0.23).

#### ROC Curves Examining Applicability of PAUSS Total in Predicting ASC Group Membership vs TC Group Membership

Kästner et al, report an AUC for the PAUSS of .82, suggesting good ability in discriminating ASC diagnosis compared to a disease-control sample, and proposed a cut-off PAUSS score of 15 in order to achieve 72.3% sensitivity and 71.1% specificity. Within our sample, in predicting ASC group membership vs TC group membership, we found an excellent predictive ability of the PAUSS (AUC = 0.926, Std.Error = 0.02, *P* < .001, 95% CI = 0.887–0.965), with a suggested cut-off of 10 for 88.3% sensitivity and 93.6 specificity.

### Aim Two: To Investigate the Short and Long-term Stability of the PAUSS

#### Short-term Stability of the PAUSS Within SSD Cohort

366 Participants within the SCOPE SSD cohort provided both baseline and 2-week follow-up data. Correlational analyses found fair test–retest reliability .4 to .59^35^ for the majority of individual PAUSS items (N3: *r* = .45, *P* < .001; N4: *r* = .59, *P* < .001; N6: *r* = .49, *P* < .001; G5: *r* = .56, *P* < .001; G15: *r* = .52, *P* < .001). Additionally, good test–retest reliability (.60 to .74^35^) was found for N1 (*r* = .72, *P* < .001), N5 (*r* = .70, *P* < .001), N7 (*r* = .68, *P* < .001), and PAUSS total (*r* = .65, *P* < .001).

### Long-term Stability of the PAUSS Within SSD Cohort

716 Participants within the GROUP SSD cohort provided both baseline and 3-year follow-up data, with 583 participants providing both baseline and 6-year follow-up data. Test–retest reliability was found to be poor (below 0.4^35^) for all individual PAUSS items except for 3-year N5 (*r* = .45, *P* < .001) which was fair. PAUSS total was found to have fair test–retest reliability at both 3- (*r* = .485, *P* < .001) and 6-year (*r* = .446, *P* < .001) follow-up.

## Discussion

The PAUSS has become an increasingly popular measure of autistic traits in populations with psychosis because it is easily derived from a commonly used clinical assessment, the PANSS. Although initial PAUSS evaluation was encouraging, it has received little external validation, particularly in large sample studies, nor has it been examined longitudinally. First, we investigated the conceptual replicability of the original validation,^13^ extending this into related populations. Second, we examined the PAUSS long-term stability. Results differed in important ways from the original validation.^13^ SSD-siblings and TC had significantly lower PAUSS scores than SSD and ASC cohorts. Cronbach’s alpha was acceptable for the SSD cohort only. Intercorrelations of the PAUSS total and ADOS total, and of individual PAUSS items with the ADOS total, generally replicated in a similar, albeit weaker, direction to Kästner et al’s original findings.

Although the SSD cohort’s Cronbach’s alpha showed good internal consistency of the PAUSS, the ASC and TC cohorts indicated unacceptable internal consistency. Notably, the low Cronbach’s alphas were driven by several negative inter-item correlations. As the PANSS, which the PAUSS is derived from, is not intended for use within non-psychotic populations, this is not unexpected, however, it is of relevance to researchers wishing to measure or compare autistic traits across multiple diagnostic groups.

It is notable that SSD participants, both within our dataset, and within Kästner et al,^13^ reported higher PAUSS scores than ASC participants. Surprisingly, none of our ASC participants met Kästner et al’s proposed cut-off of 30 for PAUSS-Autistic, and very few met the revised cut-off of 24 developed from our SSD dataset PAUSS percentiles. ROC analysis indicated that PAUSS scores of >10 discriminated ASC from TC participants with excellent sensitivity and specificity. Kästner et al’s percentile-based cut-off of 30 for PAUSS-Autistic was much higher than the cut-off of 15 indicated by their ROC curve. This was replicated in our own findings, with a higher percentile cut-off and lower ROC cut-off. Distinct cut-offs are not necessarily problematic and may indicate, for example, that ASC classification may be appropriate above a certain score, with below a different score indicating that ASC classification is unlikely. However, our findings suggest that the proposed PAUSS-Autistic cut-offs need revision.

It is also notable that over 10% of our ASC cohort were classified as PAUSS-non-Autistic even with our revised cut-offs. Additionally, because very few ASC participants were classified as PAUSS-Autistic, it may be the case that the PAUSS is substantially influenced by state-like psychotic symptoms (which would be higher within an SSD cohort) rather than autistic traits (which would be higher within an ASC cohort). As a measure of autism which is derived exclusively from a measure of psychosis, the philosophy behind the PAUSS is that certain symptoms of psychosis are the same as an autistic phenotype. If this is the case, then the findings of this study and Kästner et al’s would suggest that this autistic phenotype is stronger within SSD cohorts than ASC cohorts. Given that ASC cohorts *all* experience autistic phenotypes, whereas SSD cohorts do not, this is a problematic conclusion.

It is possible a distinct “autistic-schizophrenia” subgroup exists, who may represent a particularly severe group in terms of both psychotic symptomatology and autistic traits. If so, SSD scores may be higher than ASC due to this severe subpopulation. However, if this were occurring within the SSD population, we might predict a bimodal distribution with the majority of SSD participants within one peak of lower PAUSS scores and a separate group of higher PAUSS scorers. Our SSD sample, like Kästner et al’s, displayed a positive skew, with most participants scoring low on the PAUSS, tailing off to a small number rated as PAUSS-Autistic.

A substantial body of research suggests differences in the maintenance of ASC traits compared to SSD symptoms over time. Autism is highly stable,^23,24^ both in terms of diagnosis,^36–38^ and individual trait profiles.^39^ In contrast, SSDs are much less stable; although negative psychotic symptoms show more stability than positive symptoms, approximately 73% of individuals show a change in negative symptoms over time,^26^ particularly when assessed at the first episode.

PAUSS total exhibited good 2-week stability and fair 3- and 6-year stability according to Cicchetti’s^35^ definitions of test–retest reliability. Portney and Watkins^40^recommend more conservative test–retest reliability requirements for clinical measurements, with values from 0.75 to 0.9 acceptable and values over 0.9 good. This may be a more appropriate standard for autistic traits, where true change is proposed to be low. This pattern of stability therefore appears more reflective of psychotic symptoms than autistic traits, and is notable as it suggests that, in contrast to the PAUSS’s aims, negative symptoms of psychosis are not akin to traits seen within autism. The PANSS-6^41^ assesses psychotic symptom severity and post-treatment changes, and includes three PAUSS questions (N1, N4, N6). Given the lack of stability, the PAUSS may be a proxy for negative symptom severity or impairment, rather than autistic characteristics.

More broadly, certain PANSS items which are included within the PAUSS represent qualities that were historically characterized as autistic but have more recently been challenged by emerging research and autistic scholarship.^42^ For example, “Poor Rapport” (N3) focuses on a “lack of interpersonal empathy.” Recent work suggests that autistic people often report high rapport with specific partners, especially other autistic people, challenging the notion that they lack capacity for social connection.^43,44^ While autistic individuals may not display non-autistic markers of empathy and may misunderstand non-autistic social norms, they still feel empathy acutely^45^ and misunderstandings are bidirectional: non-autistic individuals also often misinterpret autistic social expressions and behaviors.^46–48^

Results should be interpreted considering the following strengths and limitations. This is the first study to investigate the PAUSS longitudinally, and to include both SSD-siblings and TCs. The large and diverse sample from two distinct datasets also represents a strength. Our SSD sample had much higher negative and general psychotic symptoms than Kästner et al‘s SSD sample, while positive psychotic symptoms were comparable. Our SSD sample also represent a broader age range than the original sample. Sample differences may partly explain the difference in findings. However, for the PAUSS to be a valuable research instrument it needs to demonstrate validity and reliability across different levels of psychotic illness severity. Whilst Kästner et al. refer to “autism behavioural clusters,” and group the PAUSS items into those related to “differences in social interaction,” “differences in communication,” and “restricted and repetitive patterns of behaviour,” no data reduction analysis (e.g., EFA, PCA) was performed on the PAUSS items. This may explain the present study’s relatively low Cronbach’s alphas, and future measure development aiming to assess autism within SSD cohorts should undertake appropriate data reduction techniques. Our assessment of ASC vs TC for the ROC curves may have distinguished psychopathology present versus absent rather than ASC present versus absent since the groups are likely too disparate to produce a meaningful cut-off. Our critical *P*-value value was set at .005, rather than the .05 used in Kästner et al’s original paper, however, this difference in alpha value did not change the overall interpretation of analyses. Finally, our conceptual replication was limited by our available dataset, preventing us from replicating all of Kästner et al’s original analyses.

## Conclusion

Although the PAUSS is growing in popularity, our results suggest that the PAUSS may not be suitable for assessing autistic traits. The virtually non-existent rate of PAUSS-Autistic within ASC populations is a cause for concern, suggesting that the PAUSS may not accurately reflect the characteristics of autism. The relative lack of long-term stability of the PAUSS suggests that measuring psychotic symptoms is not akin to measuring autistic traits and that although (negative) psychotic symptoms may share similarities with autistic traits, the phenotypes are subtly different in important ways, with implications for prognosis, treatment, and etiology. Ultimately, our findings suggest that the PAUSS may be capturing something of clinical importance, but it seems likely that it does not truly capture autism. Future research is needed investigating how to accurately measure autistic traits within psychosis, potentially using more elaborate or creative assessments^49^ and modeling psychotic and autistic phenotypes.^50^ This will support the development of a fundamental understanding of the etiology of the two conditions, as well as allowing an understanding of how autistic traits within psychosis may impact upon development, treatment, and prognosis.

## Supplementary Material

Supplementary material is available at https://academic.oup.com/schizophreniabulletin/.

sbad161_suppl_Supplementary_Material
