# Supplementary material for: A Cross-sectional Conceptual Replication and Longitudinal Evaluation of the PANSS-Autism-Severity-Score Measure Suggests it Does Not Capture Autistic Traits in Individuals With Psychosis
Source: Schizophr Bull. 2023 Nov 22;51(1):186–97. doi: 10.1093/schbul/sbad161 (PMC11661953; doi:10.1093/schbul/sbad161)
Supplement: sbad161_suppl_Supplementary_Material [file sbad161_suppl_supplementary_material.docx]

**Supplementary Materials**

**Supplement 1: Eligibility criteria, recruitment, and interrater reliability**

***S1.1: Eligibility criteria***

*GROUP ^1^:*

Schizophrenia Spectrum Disorder (SSD): (1) age 16 to 50 years; (2) diagnosis of DSM-IV non-affective psychotic disorder; (3) Dutch language speaker; (4) capacity to consent.

SSD-Siblings: (1) age 16 to 50 years; (2) Dutch language speaker; (3) capacity to consent.

Typical control (TC): (1) age 16 to 50 years; (2) no lifetime psychotic disorder; (3) no first degree family member with a lifetime psychotic disorder; (4) Dutch language speaker; (5) capacity to consent.

*SCOPE ^2,3^:*

SSD: Inclusion; (1) diagnosis of DSM-IV schizophrenia or schizoaffective disorder; (2) no hospitalisations within past two months; (3) stable medication regimen for a minimum of 6 weeks with no dose changes for a minimum of 2 weeks; (4) age 18-65 years.

Exclusion; (1) presence or history of pervasive developmental disorder or IQ < 70; (2) presence or history of medical or neurological disorders that may affect brain function; (3) presence of sensory limitation including visual or hearing impairments that interfere with assessment; (4) lack of English proficiency; (5) substance abuse in past month; (6) presence of substance dependence not in remission for the past 6 months; (7) age 18-65 years.

*SCOPE-A ^4,5^:*

Autism Spectrum Condition (ASC): (1) ASC diagnosis; (2) age 18-59 years.

***S1.2: Recruitment***

*GROUP ^1^:*

SSD: Patients who met eligibility criteria from geographically representative areas of the Netherlands and Belgium were identified via caseloads of clinicians working in regional psychosis departments or academic centres.

SSD-Siblings: SSD participants were asked for consent for the study team to contact their first-degree family members to take part in the study.

TC: Participants were identified via random mail outs to addresses within the same catchment area as SSD participants.

GROUP data release 7.0 was used for current analyses. GROUP was approved by the Medical Ethics Committee of the University Medical Center Utrecht, with each GROUP site subsequently gaining ethical approval from their local ethics review panel.

*SCOPE ^2,3^:*

SSD: Participants were recruited via four sites: Southern Methodist University (SMU); The University of Miami Miller School of Medicine (UM); The University of Texas at Dallas (UTD); and The University of North Carolina at Chapel Hill (UNC).

At the SMU and UTD site participants were recruited via Metrocare Services, a nonprofit mental health services provider. At the UM site patients were recruited via the Miami VA Medical Center and the Jackson Memorial Hospital-University of Miami Medical Center. UNC participants were recruited via the Schizophrenia Treatment and Evaluation Program (STEP) in Carrboro, NC, and the Clinical Research Unit (CRU) in Raleigh, NC.

SCOPE consisted of data collection across multiple phases and sites. The current study utilized data from Phases 3-5 of SCOPE’s recruitment phases, as well as the 2-week PANSS follow up data.

*SCOPE-A ^4,5^:*

ASC: ASC participants were recruited via the nonPareil Institute, a local nonprofit organization, and from the local and university community.

***S1.3: Interrater reliability***

GROUP: Interviewers interrater reliability on the PANSS was assessed via four recorded interviews, which were rated by 16 randomly selected interviewers: ICC PANSS positive subscale = 0.957; ICC PANSS negative subscale =0.911; ICC PANSS total =0.946

SCOPE and SCOPE-A: Raters were trained to adequate reliability (ICC>.80) with a gold-standard rater.

**Supplement 2: Analyses comparing SSD participants from the GROUP and SCOPE datasets**

SSD participants from GROUP and SCOPE were found to differ significantly in terms of their age (GROUPmedian=26, SCOPEmedian=40, U=343471.00, p<0.001), gender (GROUPmale=77.2%, SCOPEmale=67.6%, X^2^(1,1448)=14.53, p<0.001), PANSS total (GROUPmedian=52, SCOPEmedian=63, U=277007.50, z=9.84, p<0.001), PANSS positive (GROUPmedian=11, SCOPEmedian=16, U=302118.00, z=12.27, p<0.001), PANSS negative (GROUPmedian=13, SCOPEmedian=13, U=232652.00, z=2.07, p=0.04), and PANSS general (GROUPmedian=26, SCOPEmedian=32, U=278690.00, z=9.58, p<0.001).

**Supplement 3: Analyses examining GROUP and SCOPE SSD datasets separately**

Combining two diverse cohorts was seen as a strength of the analysis in aiding generalisability. For completeness, however, additional analyses including site (GROUP/SCOPE) as a covariate to analyses where possible, or analysing GROUP and SCOPE SSD participants separately to each other where inclusion of site covariates was not possible, can be viewed below.

S3.1 One sample t test comparing PANSS illness severity between our sample and the sample reported in Kästner et al. (2015; data provided by authors); analysis run separately for GROUP and SCOPE

Table S3.1: Differences in psychotic symptoms between the GROUP and SCOPE SSD samples with the SSD sample reported in Kästner et al. (2015)

|  | **GROUP Dataset (n=1022) mean (SD)** | **Kästner et al. (n=** **1156) Dataset** | **Significance** |
| --- | --- | --- | --- |
| **PANSS positive** | 12.69 (5.31) | 13.64 (6.24) | <0.001 |
| **PANSS negative** | 13.90 (5.90) | 18.09 (7.93) | <0.001 |
| **PANSS general** | 27.89 (8.40) | 35.04 (11.82) | <0.001 |
| **PANSS total** | 54.41 (16.80) | 66.79 (23.23) | <0.001 |
|  | **SCOPE Dataset (n=426) mean (SD)** | **Kästner et al. (n=** **1156) Dataset** | **Significance** |
| **PANSS positive** | 16.37 (5.27) | 13.64 (6.24) | <0.001 |
| **PANSS negative** | 14.29 (5.31) | 18.09 (7.93) | <0.001 |
| **PANSS general** | 32.20 (7.98) | 35.04 (11.82) | <0.001 |
| **PANSS total** | 62.86 (14.62) | 66.79 (23.23) | <0.001 |

S3.2: The internal consistency of the PAUSS items in SSD (analysis run separately for GROUP and SCOPE)

Table S3.2.1: Internal consistency of the PAUSS across cohorts

| Cohort | Cronbach’s α |
| --- | --- |
| Kästner et al. SSD | 0.86 |
| GROUP SSD | 0.80 |
| SCOPE SSD | 0.67 |

Table S3.2.2: Item to item Spearman’s rho intercorrelation matrix for individual PAUSS items for all cohorts (site added as covariate)

|  | Blunted Affect (PANSS N1) | Poor rapport (PANSS N3) | Social withdrawal (PANSS N4) | Abstract thinking (PANSS N5) | Lack of spontaneity (PANSS N6) | Stereotyped thinking (PANSS N7) | Mannerism (PANSS G5) |
| --- | --- | --- | --- | --- | --- | --- | --- |
| Poor rapport (PANSS N3) | | | | | | | |
| Kästner et al. SSD | **0.668** | - |  |  |  |  |  |
| SSD | **0.56** | - |  |  |  |  |  |
| Social withdrawal (PANSS N4) | | | | | | | |
| Kästner et al. SSD | **0.574** | **0.577** | - |  |  |  |  |
| SSD | **0.45** | **0.43** | - |  |  |  |  |
| Abstract thinking (PANSS N5) | | | | | | | |
| Kästner et al. SSD | **0.472** | **0.452** | 0.303 | - |  |  |  |
| SSD | 0.21 | 0.23 | 0.21 | - |  |  |  |
| Conversation (PANSS N6) | | | | | | | |
| Kästner et al. SSD | **0.577** | **0.598** | **0.573** | **0.432** | - |  |  |
| SSD | **0.61** | **0.66** | **0.42** | 0.28 | - |  |  |
| Stereotyped thinking (PANSS N7) | | | | | | | |
| Kästner et al. SSD | **0.414** | **0.442** | 0.332 | **0.400** | 0.254 | - |  |
| SSD | 0.24 | 0.31 | 0.28 | 0.26 | 0.25 | - |  |
| Mannerism (PANSS G5) | | | | | | | |
| Kästner et al. SSD | 0.263 | 0.244 | 0.218 | 0.245 | 0.137 | 0.315 | - |
| SSD | 0.32 | 0.27 | 0.17 | 0.13 | 0.27 | 0.29 | - |
| Preoccupation (PANSS G15) | | | | | | | |
| Kästner et al. SSD | **0.490** | **0.558** | **0.499** | 0.391 | 0.363 | **0.523** | 0.293 |
| SSD | 0.16 | 0.24 | 0.21 | 0.16 | 0.15 | **0.50** | 0.22 |
| Correlation coefficients ≥ 0.4 are set in bold.   - SSD (n=1448): All correlations were significant at the p<0.001 level | | | | | | | |

S3.3: The number of SSD participants from GROUP and SCOPE datasets falling into Kästner et al.’s defined cut offs (based on their 1^st^ and last PAUSS total percentile) for PAUSS ‘autistic’ and ‘non-autistic’ were calculated, and ‘autistic’ and ‘non-autistic’ were then compared on participant characteristics and PANSS scores using Mann Whitney U tests for scale data and Fishers Exact test for gender; analysis run separately for GROUP and SCOPE

Additionally, 1^st^ and last PAUSS total percentile based on our dataset was computed, and again, ‘autistic’ and ‘non-autistic’ were compared on participant characteristics and PANSS scores. PAUSS items we’re excluded when calculating all PANSS related items; analysis run separately for GROUP and SCOPE

Table S3.3: Comparison of PAUSS autistic and non-autistic based on Kästner et al.’s original cut-offs (‘NonAutistic-Schizophrenia’; PAUSS total≤10 and ‘Autistic-Schizophrenia’; PAUSS total≥30) and alternative cut-offs derived from our combined SSD cohort (8 for PAUSS ‘non-autistic’ and ≥24 for PAUSS ‘autistic’)

|  | PAUSS ‘autistic’ | PAUSS ‘non-autistic’ | Sig |
| --- | --- | --- | --- |
| **Original cut-offs** | | | |
| Kästner et al. SSD n (%) | 137 (11.85%) of 1156 | 168 (14.53%) of 1156 | - |
| GROUP dataset n (%) | 15 (1.5%) of 1022 | 316 (30.9%) of 1022 | - |
| SCOPE dataset n (%) | 6 (1.4%) of 426 | 74 (17.4%) of 426 | - |
| GROUP Age mean/median (SD) | 24.20 / 24 (3.28) | 27.79 / 26 (7.83) | 0.1 |
| SCOPE Age mean/median (SD) | 34.83 / 28.50 (15.30) | 39.97 / 40.50 (11.37) | 0.2 |
| GROUP Gender male n (%) | 14 (93.33) | 222 (70.25) | 0.08 |
| SCOPE Gender male n (%) | 6 (100.00) | 46 (62.16) | 0.9 |
| GROUP PANSS Total (PAUSS items excluded) mean/median (SD) | 62.54 / 66 (13.40) | 30.90 / 29 (7.03) | <0.0001 |
| SCOPE PANSS Total (PAUSS items excluded) mean/median (SD) | 58.50 / 60.50 (10.63) | 39.97 / 40.50 (10.12) | 0.002 |
| GROUP PANSS positive mean/median (SD) | 17.07 / 17 (7.29) | 10.16 / 9 (3.44) | <0.0001 |
| SCOPE PANSS positive mean/median (SD) | 17.50 / 16 (4.68) | 14.14 / 14 (5.41) | 0.1 |
| GROUP PANSS negative (PAUSS items excluded) mean/median (SD) | 4.40 / 5 (1.40) | 1.23 / 1 (0.52) | <0.0001 |
| SCOPE PANSS negative (PAUSS items excluded) mean/median (SD) | 3.33 / 3.5 (1.37) | 1.11 / 1 (0.48) | <0.0001 |
| GROUP PANSS general (PAUSS items excluded) mean/median (SD) | 39.85 / 43 (8.45) | 19.55 / 19 (4.42) | <0.0001 |
| SCOPE PANSS general (PAUSS items excluded) mean/median (SD) | 37.67 / 38 (8.12) | 24.73 / 23.50 (6.49) | 0.001 |
| **Alternative cut-offs** | | | |
| GROUP dataset n (%) | 111 (10.9%) of 1022 | 158 (15.5%) of 1022 | - |
| SCOPE dataset n (%) | 40 (9.4%) of 426 | 24 (5.6%) of 426 | - |
| GROUP Age mean/median (SD) | 25.90 / 24 (7.59) | 28.32 / 26 (8.32) | 0.004 |
| SCOPE Age mean/median (SD) | 37.15 / 36 (11.94) | 40.04 / 38 (12.20) | 0.4 |
| GROUP Gender male n (%) | 93 (83.8) | 104 (65.82) | 0.001 |
| SCOPE Gender male n (%) | 30 (75.0) | 15 (62.5) | 0.4 |
| GROUP PANSS Total (PAUSS items excluded) mean/median (SD) | 53.80 / 54 (12.88) | 28.66 / 27 (5.58) | <0.0001 |
| SCOPE PANSS Total (PAUSS items excluded) mean/median (SD) | 55.93 / 54.50 (11.47) | 42.00 / 45.50 (11.68) | <0.0001 |
| GROUP PANSS positive mean/median (SD) | 16.72 / 16 (6.48) | 9.39 / 9 (2.86) | <0.0001 |
| SCOPE PANSS positive mean/median (SD) | 17.95 / 16.50 (6.10) | 14.67 / 15 (5.99) | 0.09 |
| GROUP PANSS negative (PAUSS items excluded) mean/median (SD) | 3.64 / 4 (1.10) | 1.04 / 1 (0.24) | <0.0001 |
| SCOPE PANSS negative (PAUSS items excluded) mean/median (SD) | 3.30 / 3.50 (1.24) | 1.08 / 1 (0.41) | <0.0001 |
| GROUP PANSS general (PAUSS items excluded) mean/median (SD) | 34.28 / 33 (7.74) | 18.30 / 18 (3.83) | <0.0001 |
| SCOPE PANSS general (PAUSS items excluded) mean/median (SD) | 34.68 / 34.50 (6.52) | 26.25 / 27 (6.92) | <0.0001 |

**Supplement 4: Analyses including age and sex as covariates**

In line with Kästner et al., age and gender were not included as covariates in the main analysis. Analyses including age and sex as covariates, where possible, can be viewed below. Where age and sex cannot be included in an analysis as covariates, males and females are analysed separately.

S4.1 The internal consistency, via Cronbach’s α and inter-item correlations, of the PAUSS items in all participant groups

Cronbach’s α can be seen in table S41.1. Interitem correlations in table S4.1.2

Table S4.1.1 : Internal consistency of the PAUSS across cohorts, by gender

| Cohort | Cronbach’s α |
| --- | --- |
| Kästner et al. SSD | 0.86 |
| Male SSD | 0.76 |
| Female SSD | 0.74 |
| Male ASC | 0.57 |
| Female ASC | 0.66 |
| Male SSD-sibling | 0.79 |
| Female SSD-sibling | 0.48 |
| Male TC | 0.40 |
| Female TC | 0.73 |

Table S4.1.2: Item-to-item Spearman’s rho intercorrelation matrix for individual PAUSS items for all cohorts and Spearman’s rho intercorrelations of individual PAUSS items with the ADOS total, in the ASC sample (age and sex added as covariates)

|  | Blunted Affect (N1) | Poor rapport (N3) | Social withdrawal (N4) | Abstract thinking (N5) | Lack of spontaneity (N6) | Stereotyped thinking (N7) | Mannerism (G5) | Preoccupation (G15) |
| --- | --- | --- | --- | --- | --- | --- | --- | --- |
| Poor rapport (PANSS N3) | | | | | | | |  |
| Kästner et al. SSD | **0.67** | - |  |  |  |  |  |  |
| SSD | **0.55** | - |  |  |  |  |  |  |
| ASC | **0.52** | - |  |  |  |  |  |  |
| SSD-siblings | **0.50** | - |  |  |  |  |  |  |
| TC | -0.03 | - |  |  |  |  |  |  |
| Social withdrawal (PANSS N4) | | | | | | | |  |
| Kästner et al. SSD | **0.57** | **0.58** | - |  |  |  |  |  |
| SSD | **0.45** | **0.42** | - |  |  |  |  |  |
| ASC | **0.45** | 0.28 | - |  |  |  |  |  |
| SSD-siblings | **0.48** | 0.34 | - |  |  |  |  |  |
| TC | -0.02 | 0.16 | - |  |  |  |  |  |
| Abstract thinking (PANSS N5) | | | | | | | |  |
| Kästner et al. SSD | **0.47** | **0.45** | 0.303 | - |  |  |  |  |
| SSD | 0.19 | 0.18 | 0.21 | - |  |  |  |  |
| ASC | 0.22 | 0.18 | 0.15 | - |  |  |  |  |
| SSD-siblings | 0.18 | 0.18 | 0.09 | - |  |  |  |  |
| TC | -0.05 | 0.04 | -0.03 | - |  |  |  |  |
| Conversation (PANSS N6) | | | | | | | |  |
| Kästner et al. SSD | **0.58** | **0.60** | **0.57** | **0.43** | - |  |  |  |
| SSD | **0.60** | **0.65** | **0.42** | 0.26 | - |  |  |  |
| ASC | **0.51** | **0.50** | 0.37 | 0.13 | - |  |  |  |
| SSD-siblings | **0.55** | **0.57** | **0.42** | 0.18 | - |  |  |  |
| TC | 0.18 | 0.10 | 0.12 | 0.21 | - |  |  |  |
| Stereotyped thinking (PANSS N7) | | | | | | | |  |
| Kästner et al. SSD | **0.41** | **0.44** | 0.33 | **0.40** | 0.25 | - |  |  |
| SSD | 0.24 | 0.30 | 0.28 | 0.26 | 0.25 | - |  |  |
| ASC | -0.008 | -0.02 | 0.03 | 0.24 | 0.04 | - |  |  |
| SSD-siblings | 0.24 | 0.27 | 0.32 | 0.19 | 0.26 | - |  |  |
| TC | -0.02 | 0.19 | 0.20 | -0.03 | 0.15 | - |  |  |
| Mannerism (PANSS G5) | | | | | | | |  |
| Kästner et al. SSD | 0.26 | 0.24 | 0.22 | 0.25 | 0.14 | 0.32 | - |  |
| SSD | 0.30 | 0.25 | 0.17 | 0.16 | 0.25 | 0.29 | - |  |
| ASC | -0.02 | 0.15 | -0.10 | 0.05 | -0.09 | -0.11 | - |  |
| SSD-siblings | 0.23 | 0.32 | 0.36 | 0.09 | 0.30 | 0.26 | - |  |
| TC | -0.02 | 0.20 | -0.01 | -0.03 | -0.02 | -0.01 | - |  |
| Preoccupation (PANSS G15) | | | | | | | |  |
| Kästner et al. SSD | **0.49** | **0.56** | **0.50** | 0.39 | 0.36 | **0.52** | 0.29 | - |
| SSD | 0.16 | 0.24 | 0.21 | 0.15 | 0.15 | **0.49** | 0.21 | - |
| ASC | -0.15 | -0.15 | -0.12 | 0.10 | -0.15 | **0.40** | 0.09 | - |
| SSD-siblings | 0.20 | 0.24 | 0.28 | 0.04 | 0.35 | 0.24 | 0.10 | - |
| TC | 0.12 | 0.16 | 0.18 | -0.04 | 0.13 | **0.67** | -0.01 | - |
| ADOS (ASC cohort only) | | | | | | | |  |
| Kästner et al. ASC | **0.6-0.7** | **0.5-0.6** | **0.6-0.7** | **0.4-0.5** | **0.6-0.7** | **0.4-0.5** | **0.6-0.7** | **0.6-0.7** |
| ASC | 0.39 | 0.29 | **0.43** | 0.29 | **0.40** | 0.19 | -0.04 | 0.01 |
| Correlation coefficients ≥ 0.4 are set in bold.   - SSD (n=1448): All correlations were significant at the p<0.001 level - For ASC (n=103): Correlations >0.27 were significant at the p<0.005 level, and all correlations >0.36 were significant at the p<0.001 level - For SSD-siblings (n=700): Correlations >0.11 were significant at the p<0.005 level, and all correlations >0.17 were significant at the p<0.001 level - For TC (n=409): Correlations >0.14 were significant at the p<0.005 level, and all correlations >0.16 were significant at the p<0.001 level   Abbreviations: Positive and Negative Syndrome Scale (PANSS); Schizophrenia Spectrum Disorder (SSD); Autism Diagnostic Observation Schedule (ADOS); Autism Spectrum Condition (ASC); Typical Control (TC) | | | | | | | | |

S4.2: The convergent validity, via spearman’s rho intercorrelations, of the PAUSS total with ADOS total, age, and WASI IQ, as well as intercorrelations of individual PAUSS items with the ADOS total, in the ASC sample.

Intercorrelations of individual PAUSS items with the ADOS total, in the ASC sample, can be viewed in Table S4.1.2 (age and sex added as covariates)

Spearmans rho intercorrelations, of the PAUSS total with ADOS total, age, and WASI IQ:

PAUSS – ADOS (age and sex added as covariates) r_2_ = - 0.52, p <0.001

PAUSS – WASI IQ (age and sex added as covariates) r_2_ = - 0.20, p =0.045

PAUSS – Age (sex added as a covariate) r_2_ = - -0.11, p =0.3

S4.3 PAUSS-Autistic and PAUSS-Non-Autistic SSD participants compared on participant characteristics and PANSS scores, via a series of Mann-Whitney U and Chi-Square tests.

Table S4.3 Comparison of PAUSS autistic and non-autistic based on Kästner et al.’s original cut-offs (‘NonAutistic-Schizophrenia’; PAUSS total≤10 and ‘Autistic-Schizophrenia’; PAUSS total≥30) and alternative cut-offs derived from our combined SSD cohort (8 for PAUSS ‘non-autistic’ and ≥24 for PAUSS ‘autistic’). Gender analysed separately. As there was only 1 female participant characterised as PAUSS ‘autistic’ using Kästner et al.’s original cut-offs, results for male participants only are presented.

|  | PAUSS ‘autistic’ | PAUSS ‘non-autistic’ | Sig |
| --- | --- | --- | --- |
| **Original cut-offs** | | | |
| Kästner et al. SSD n (%) | 137 (11.85%) of 1156 | 168 (14.53%) of 1156 | - |
| Male n (%) | 20 (1.9%) of 1077 | 268 (24.9%) of 1077 | - |
| Female n (%) | 1 (0.3%) of 371 | 122 (32.9%) of 371 | - |
| Male Age mean/median (SD) | 27.30 / 25 (9.75) | 28.90 / 26 (9.31) | 0.2 |
| Male PANSS Total (PAUSS items excluded) mean/median (SD) | 60.56/ 66 (12.41) | 32.51 / 31 (7.94) | <0.0001 |
| Male PANSS positive mean/median (SD) | 16.80 / 16.5 (6.45) | 11.06 / 10 (4.13) | 0.008 |
| Male PANSS negative (PAUSS items excluded) mean/median (SD) | 4.10 / 4 (1.48) | 1.20 / 1 (0.52) | <0.0001 |
| Male PANSS general (PAUSS items excluded) mean/median (SD) | 38.83 / 41.5 (8.30) | 20.28 / 20 (4.80) | <0.0001 |
| **Alternative cut-offs** | | | |
| Male n (%) | 123 (11.4%) of 1077 | 119 (11%) of 1077 | - |
| Female dataset n (%) | 28 (7.5%) of 371 | 63 (17%) of 371 | - |
| Male Age mean/median (SD) | 28.43 / 25 (9.58) | 29.04 / 26 (9.20) | 0.02 |
| Female Age mean/median (SD) | 30.86 / 26.50 (12.59) | 31.41 / 28 (10.58) | 0.7 |
| Male PANSS Total (PAUSS items excluded) mean/median (SD) | 55.67 / 55 (12.02) | 30.25 / 28 (7.52) | <0.001 |
| Female PANSS Total (PAUSS items excluded) mean/median (SD) | 52.71 / 49.50 (14.26) | 30.89 / 28.5 (9.16) | <0.001 |
| Male PANSS positive mean/median (SD) | 17.18 / 17 (6.27) | 10.19 / 9 (3.83) | <0.001 |
| Female PANSS positive mean/median (SD) | 16.46 / 15.50 (6.94) | 9.90 / 8 (3.95) | <0.001 |
| Male PANSS negative (PAUSS items excluded) mean/median (SD) | 3.62 / 4 (1.18) | 1.05 / 1 (0.26) | <0.001 |
| Female PANSS negative (PAUSS items excluded) mean/median (SD) | 3.25 / 3 (0.97) | 1.05 / 1 (0.28) | <0.001 |
| Male PANSS general (PAUSS items excluded) mean/median (SD) | 34.71 / 34 (7.17) | 19.08 / 18 (4.66) | <0.001 |
| Female PANSS general (PAUSS items excluded) mean/median (SD) | 33 / 32 (8.40) | 19.94 / 18 (5.92) | <0.001 |

S4.4 Comparison of PAUSS score between the ADOS groups (autism>11/autism spectrum 7-11) within the ASC sample was examined via a Mann-Whitney U test. Analysed separately by gender.

Male:

Within our male ASC cohort 31 participants were classified by the ADOS as autistic (PAUSS mean=16.39, median=17, SD=4.55), 60 as autistic spectrum (PAUSS mean=12.58, median=12, SD=3.32), 1 had missing ADOS data, and no participants fell into the no autism category. PAUSS score of the two ADOS groups differed significantly (p<0.001, η^2^=0.17).

Female:

Within our female ASC cohort 5 participants were classified by the ADOS as autistic (PAUSS mean=18.40, median=18, SD=2.70), 5 as autistic spectrum (PAUSS mean=9.2, median=8, SD=1.79), 1 had missing ADOS data, and no participants fell into the no autism category. Due to the small sample size, statistical analyses were not run to investigate significance of PAUSS total score differences between the two ADOS groups.

S4.5 The ability of the PAUSS to predict ASC group membership compared to TC group membership was investigated via receiver operator curve (ROC) analysis. Gender analysed separately.

Male:

Within our male sample there were 92 ASC participants and 186 TC. We found an excellent predictive ability of the PAUSS (AUC = 0.93, Std.Error = 0.02, p<0.001, 95%CI= 0.891-0.970) in predicting ASC group membership vs TC group membership, with a suggested cut-off of 10 for 90.2% sensitivity and 96.2 specificity.

Female:

Within our female sample there were 11 ASC participants and 223 TC. We found an excellent predictive ability of the PAUSS (AUC = 0.85, Std.Error = 0.08, p<0.001, 95%CI= 0.681-1.012) in predicting ASC group membership vs TC group membership, with a suggested cut-off of 10 for 72.7% sensitivity and 99.1 specificity.

S4.4 To address the second aim of the paper investigating the short- and long-term stability of the PAUSS, short (2-week) and long-term (3- and 6-year) test-retest reliability of the PAUSS in SSD participants was examined via Pearsons r correlations (age and sex added as covariates)

***Short term stability of the PAUSS within SSD cohort:***

366 participants within the SCOPE SSD cohort provided both baseline and two-week follow-up data, as well as data on gender and age. Correlational analyses found fair test-retest reliability 0.4 to 0.59 ^35^ for the majority of individual PAUSS items (N3: r= 0.45, p<0.001; N6: r= 0.48, p<0.001; G5: r= 0.56, p<0.001; G15: r= 0.53, p<0.001). Additionally, good test-retest reliability (0.60 to 0.74 ^35^) was found for N1 (r= 0.72, p<0.001), N4: r= 0.60, p<0.001, N5 (r= 0.70, p<0.001), N7 (r= 0.68, p<0.001), and PAUSS total (r= 0.66, p<0.001).

***Long-term stability of the PAUSS within SSD cohort:***

713 participants within the GROUP SSD cohort provided both baseline and three-year follow-up data as well as data on gender and age. 580 participants provided both baseline and six-year follow-up data, as well as data on gender and age. Test-retest reliability was found to be poor (below 0.4 ^35^) for all individual PAUSS items except for three-year N1 (r=0.40, p<0.001) and N5 (r= 0.44, p<0.001) which were fair. PAUSS total was found to have fair test-retest reliability at both three- (r= 0.47, p<0.001) and six-year (r= 0.46, p<0.001) follow-up.

**References:**

1. Korver N, Quee PJ, Boos HB, Simons CJ, de Haan L, Investigators G. Genetic Risk and Outcome of Psychosis (GROUP), a multi site longitudinal cohort study focused on gene–environment interaction: objectives, sample characteristics, recruitment and assessment methods. *International journal of methods in psychiatric research.* 2012;21(3):205-221.

2. Pinkham AE, Penn DL, Green MF, Harvey PD. Social cognition psychometric evaluation: Results of the initial psychometric study. *Schizophrenia bulletin.* 2016;42(2):494-504.

3. Pinkham AE, Harvey PD, Penn DL. Social cognition psychometric evaluation: results of the final validation study. *Schizophrenia Bulletin.* 2018;44(4):737-748.

4. Morrison KE, Pinkham AE, Kelsven S, Ludwig K, Penn DL, Sasson NJ. Psychometric evaluation of social cognitive measures for adults with autism. *Autism Research.* 2019;12(5):766-778.

5. Pinkham AE, Morrison KE, Penn DL, et al. Comprehensive comparison of social cognitive performance in autism spectrum disorder and schizophrenia. *Psychological Medicine.* 2020;50(15):2557-2565.
